# *In Vivo* Effects of Vanadium Pentoxide and Antioxidants (Ascorbic Acid and Alpha-Tocopherol) on Apoptotic, Cytotoxic, and Genotoxic Damage in Peripheral Blood of Mice

**DOI:** 10.1155/2016/6797851

**Published:** 2016-06-19

**Authors:** María del Carmen García-Rodríguez, Lourdes Montserrat Hernández-Cortés, Mario Agustín Altamirano-Lozano

**Affiliations:** Unidad de Investigación en Genética y Toxicología Ambiental (UNIGEN), Facultad de Estudios Superiores “Zaragoza”, Universidad Nacional Autónoma de México (UNAM), P.O. Box 9-020, 15000 México, DF, Mexico

## Abstract

This study was conducted to investigate the effects of vanadium pentoxide (V_2_O_5_), ascorbic acid (AA), and alpha-tocopherol (*α*-TOH) on apoptotic, cytotoxic, and genotoxic activity. Groups of five Hsd:ICR mice were treated with the following: (a) vehicle, distilled water; (b) vehicle, corn oil; (c) AA, 100 mg/kg intraperitoneally (ip); (d) *α*-TOH, 20 mg/kg by gavage; (e) V_2_O_5_, 40 mg/kg by ip injection; (f) AA + V_2_O_5_; and (g) *α*-TOH + V_2_O_5_. Genotoxic damage was evaluated by examining micronucleated polychromatic erythrocytes (MN-PCE) obtained from the caudal vein at 0, 24, 48, and 72 h after treatments. Induction of apoptosis and cell viability were assessed at 48 h after treatment in nucleated cells of peripheral blood. Treatment with AA alone reduced basal MN-PCE, while V_2_O_5_ treatment marginally increased MN-PCE at all times after injection. Antioxidants treatments prior to V_2_O_5_ administration decreased MN-PCE compared to the V_2_O_5_ group, with the most significant effect in the AA + V_2_O_5_ group. The apoptotic cells increased with all treatments, suggesting that this process may contribute to the elimination of the cells with V_2_O_5_-induced DNA damage (MN-PCE). The necrotic cells only increased in the V_2_O_5_ group. Therefore, antioxidants such as AA and *α*-TOH can be used effectively to protect or reduce the genotoxic effects induced by vanadium compounds like V_2_O_5_.

## 1. Introduction

For several decades, vanadium was considered a low-toxicity essential trace element with antidiabetic and anticarcinogenic properties [1, 2]. However, in 2006, the International Association for Research on Cancer (IARC) classified vanadium pentoxide (V_2_O_5_) as a Group 2B substance (possibly carcinogenic to humans) based on results in experimental animals [3]. Three years later, in 2009, the American Council of Government and Industrial Hygienists (ACGIH) placed V_2_O_5_ in category A3 (confirmed animal carcinogen with unknown relevance to humans) [4]. Today, there is disagreement regarding the carcinogenic responses to V_2_O_5_ and evidence supporting that a genotoxic mode of action is still insufficient [5]. The general consensus is that while both positive and negative results have been reported, the weight of evidence suggests that V_2_O_5_ has the potential to induce aneuploidy, micronucleus (MN), and chromosomal aberrations in some cells* in vitro* and* in vivo* (somatic cells) [3, 6, 7].

Among the handful of proposed mechanisms of vanadium(V) toxicity, which include interference with protein phosphatase and kinase activity and inhibition of DNA repair, the induction of oxidative stress is of particular importance for biological systems [7–9]. The genotoxicity associated with oxidative stress is based on the oxidative mechanism of reduction of vanadium(V), generating reactive oxygen species (ROS) such as hydroxyl radicals (^•^OH) [10]. Since antioxidants are able to inactivate highly reactive molecules such as ROS that are generated during various biochemical processes in the cells [11], substances with antioxidant properties emerge as putative preventatives and coadjuvants in the treatment of chronic degenerative diseases related to oxidative stress and DNA damage [12].

A large number of antioxidants have been shown to reduce the clastogenicity of drugs and pesticides in experimental animals [13–15]; these include ascorbic acid (AA) and alpha-tocopherol (*α*-TOH). The beneficial properties of AA were highlighted by Cameron and Pauling in the 1970s, who suggested that high doses of AA (>10 g/day) cure and prevent cancer by promoting collagen synthesis [16]. However, researchers now suggest that AA actually prevents cancer by neutralizing ROS before they can damage DNA and initiate tumor growth; AA may also act as a prooxidant, helping the body's own ROS destroy early-stage tumors [17–19]. Similarly, it has been shown that *α*-TOH is effective in reducing the effects of various genotoxic compounds [20, 21]. In terms of bioavailability and bioactivity, *α*-TOH is biologically and functionally the most important and most active antioxidant of all the vitamin E isoforms in humans because it effectively minimizes oxidative stress and regulates lipid peroxidation and toxic effects of ROS in biological systems [22–24]. Since the 1960s and similarly to AA, it has been observed that dietary *α*-TOH supplementation is somewhat effective in suppressing carcinogen-induced cancers in rodents [25].

Although the antioxidants AA and *α*-TOH have shown great potential in reducing some cancers and genotoxic effects induced by different chemicals, there is no information on their effect against V_2_O_5_-induced genotoxicity, cytotoxicity, and apoptosis* in vivo*. Therefore, in this study we evaluated AA (water-soluble) and *α*-TOH (lipid-soluble) in order to identify and understand their possible beneficial effects against V_2_O_5_-induced genotoxicity and cytotoxicity using the MN technique and the analyses of apoptosis, necrosis, and cell viability in peripheral blood of Hsd:ICR mice.

## 2. Materials and Methods

### 2.1. Chemicals

The following chemical and reagent tests were obtained from Sigma Chemicals Co. (St. Louis, MO, USA): V_2_O_5_ (CAS number 1314-62-1), acridine orange (AO) (CAS number 10127-02-3), ethidium bromide (EB) (CAS number 1239-45-8), *α*-tocopherol (*α*-TOH) (CAS number 10127-02-3), and ascorbic acid (AA) (CAS number 50-81-7). The corn oil (delivery vehicle for fat-soluble compounds) also was obtained from Sigma Chemicals Co. (CAS number 8001-30-7).

### 2.2. Animals

Two- to three-month-old Hsd:ICR male mice (28–35 g) were used in the experiments. The animals were kept under controlled temperature (22°C) with a 12-12 h light-dark period (light 07:00–19:00 h). The mice had free access to food (Purina®-Mexico chow for small rodents) and water. All of the mice were obtained from Harlan at “Facultad de Química, Universidad Nacional Autónoma de México” (UNAM) and were acclimated for a two-week period. The Bioethics Committee of the “Facultad de Estudios Superiores-Zaragoza,” UNAM, approved the experimental protocols used in this study.

### 2.3. Experimental Design

The doses of AA and *α*-TOH were based on results obtained in a previous study, in which doses of 100 and 20 mg/kg of AA and *α*-TOH, respectively, did not increase MN in polychromatic erythrocytes (PCE) [26]. The V_2_O_5_ dosage was selected according to previous studies in which a 40 mg/kg dose administered intraperitoneally (ip) induced MN-PCE in the peripheral blood of mice [27].

The AA and V_2_O_5_ were prepared in solution by dissolving the dry compounds in sterile distilled water, and the *α*-TOH was dissolved in corn oil (vehicle for lipid-soluble compounds). The solutions (0.25 mL) were administered immediately after preparation of the compounds. The control groups were treated identically, using vehicle only (sterile distilled water or corn oil). The evaluation criteria and work conditions were set up according to OECD guidelines (test number 474), Food and Drug Administration (FDA) guidelines, Environmental Protection Agency (EPA) guidelines, and guidelines for the testing of chemicals specified by the Collaborative Study Group for the Micronucleus Test (CSGMT) and the Mammalian Mutagenesis Study Group of the Environmental Society of Japan (JEMS.MMS) for the short-term mouse peripheral blood micronucleus test [28, 29].

**Figure 1:**
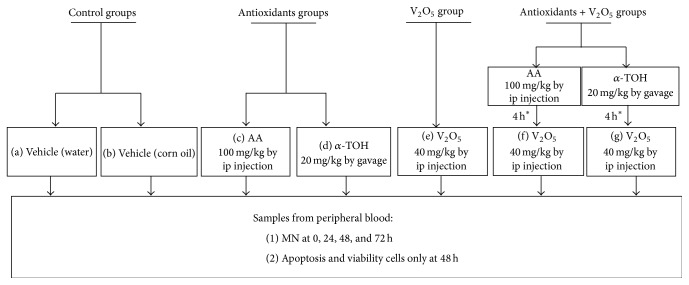
Experimental protocol. Mice were assigned at random to one of the following groups (*N* = 5 mice per group). For evaluations of MN, all animals were sampled before administering treatments (0 h) and at 24, 48, and 72 h after treatments. As for apoptosis and cell viability only at 48 h samples after treatments were taken. ^*∗*^The antioxidants were administrated 4 h before the injection of V_2_O_5_.

After establishing treatment doses, the effects of AA and *α*-TOH on genotoxic damage in V_2_O_5_-treated mice were evaluated. These assessments were performed by MN-PCE kinetic, apoptosis, and cell viability analyses. Mice were assigned randomly to one of the following groups (*N* = 5 mice per each group) according to the protocol described in Figure 1.

### 2.4. Micronucleus Assay

Slides were covered with AO and prepared in accordance with the technique described by Hayashi et al. [30]. Briefly, AO was dissolved in distilled water at a concentration of 1 mg/mL, and 10 *µ*L of this solution was placed on a preheated (approximately 70°C) clean glass slide. The AO was evenly distributed on the slide by moving a glass rod back and forth over the slide, which was then air-dried. The AO-coated glass slides were stored in a dark, dry location at room temperature prior to experimental use.

To evaluate MN after treatment, 5 *µ*L of peripheral blood samples was collected by piercing a tail blood vessel of the mice once every 24 h over a four-day period (0 to 72 h). The samples were placed directly on AO-treated slides [30]. Afterwards, a coverslip (24 × 50 mm) was placed over the sample and its edges were sealed with rubber cement. All of the prepared slides were kept in plastic boxes in the dark at 4°C. While these slide preparations cannot be stored permanently, they can be stored for several days in refrigeration if the coverslip has been sealed. Two slides were prepared for each mouse, and analyses of the slides were conducted after 12 h. The MN-PCE analysis was based on 2,000 PCE per mouse, and the presence of MN-PCE was considered genotoxic damage [29]. In parallel, the relative proportion of PCE to normochromatic erythrocytes (NCE) was analyzed in 1,000 erythrocytes.

The evaluations were made by identifying PCE, NCE, and MN-PCE under a fluorescent microscope (Nikon OPTIPHOT-2) with blue (480 nm) excitation and a barrier filter emission (515–530 nm). The differential AO staining distinguished PCE from NCE because PCE were stained, showing orange fluorescence due to the presence of ribosomal RNA, while NCE did not stain at all. The AO also allowed the identification of MN-PCE, which showed yellow fluorescence due to their DNA content. To compare the kinetics of MN-PCE induction, the data were analyzed using the “net induction frequency” (NIF) [31].

### 2.5. Apoptosis and Cell Viability Analyses

To evaluate apoptosis and cell viability, we used differential acridine orange/ethidium bromide (AO/EB) staining following the technique previously adapted for peripheral blood [12]. Blood samples (100 *µ*L) were collected by piercing a tail blood vessel of the mice prior to treatment and 48 h after treatment. Heparin (10 *µ*L) was added to the blood samples, and 20 *µ*L of AO/EB dye mix (100 *µ*L/mL AO and 100 *µ*L/mL EB, both prepared in phosphate buffered saline (PBS)) was then added. The suspension was concentrated via centrifugation (4,500 g) and the cell pellet was resuspended in 10 *µ*L of PBS and plated on a clean slide; a coverslip (24 × 24 mm) was immediately placed on the slide. Two slides for each mouse were prepared, and the analysis was conducted immediately. The apoptotic and cell viability assessments were based on 200 nucleated cells per mouse [32].

**Figure 2:**
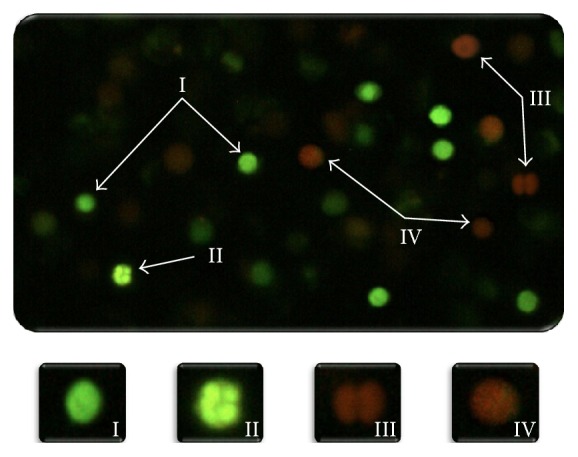
Morphology of viable cells (early apoptotic and nonapoptotic cells) and nonviable cells (late apoptotic and necrotic cells) assessed with AO/EB staining (40x). Viable cells stain uniformly showing green (I). Early apoptotic cells with intact plasma membranes appear green, with “dots” of condensed chromatin that are highly visible within (II). Late apoptotic cells are stained showing bright green-orange because membrane blebbing starts to occur. EB can enter the cells (III). Necrotic cells are stained emitting bright orange due to the entry of EB into these cells (IV).

Necrotic, apoptotic (early and late), and viable cells were identified using a fluorescent microscope (Nikon OPTIPHOT-2) with blue (480 nm) excitation and a barrier filter emission (515–530 nm) at 40x magnification. With the differential AO/EB staining, it is possible to distinguish between viable and nonviable cells based on membrane integrity. These dyes were used to identify cells that have undergone apoptosis and to distinguish between cells in the early and late stages of apoptosis, also based on membrane integrity (Figure 2). AO intercalates into the DNA, giving it a green appearance. This dye also binds to RNA, but because it cannot intercalate, the RNA stains red-orange. Thus, viable cells appear bright green. EB is only taken up by nonviable cells and also intercalates into DNA, making it appear orange. However, EB only binds weakly to RNA, which may result in a slightly red appearance. Thus, nonviable cells have bright orange nuclei because the EB overwhelms AO staining and their cytoplasm appears dark red (if any content remains). Both normal and early apoptotic nuclei in viable cells exhibit bright green fluorescence (Figure 2, I-II). In contrast, necrotic or late apoptotic nuclei in nonviable cells emit bright orange fluorescence (Figure 2, III-IV). The nuclei of viable cells with intact membranes were uniformly stained green (Figure 2, I). Early apoptotic cells with intact membranes, but in which the DNA has begun to fragment, still exhibit green nuclei because the EB cannot enter the cell, but chromatin condensation is visible as bright green patches in the nuclei (Figure 2, II). As the cell progresses through the apoptotic pathway and membrane blebbing begins to occur, EB permeates the cell, producing a green-orange stained cell (Figure 2, IV). Late apoptotic cells show bright orange patches of condensed chromatin in the nuclei; this distinguishes them from necrotic cells, which stain uniformly orange (Figure 2, III) [12, 32].

### 2.6. Statistical Analysis

The results of MN-PCE induction, the PCE/NCE ratio, the NIF of MN-PCE, the cell viability (viable/nonviable cells), and the necrotic and apoptotic cells (early/late) are expressed as the mean ± standard deviation (SD). The results from the various treatments were compared by an ANOVA/ANCOVA followed by Tukey's test. SPSS/PC V18TM software was used for the statistical analyses. For all of the analyses, *p* < 0.05 was considered to be significant.

## 3. Results

The MN-PCE averages are shown in Table 1. Although an increase of MN-PCE is observed in the control group treated with distilled water, there were no significant effects in either control group (water and corn oil vehicles). The antioxidants did not markedly affect the average MN-PCE in treated mice (Table 1). Treatment of V_2_O_5_ significantly increased the averages of MN-PCE at all times after injection, with the greatest increase at 72 h (about 4 MN-PCE). When the treatment included antioxidants (AA or *α*-TOH) and V_2_O_5_, the number of MN-PCE observed at 24, 48, and 72 h after treatment was lower than when treated with V_2_O_5_ alone.

As shown in Table 1, the baseline MN-PCE varied between groups (time 0), which obscured actual MN-PCE increases. Therefore, calculation of the NIF was performed to improve the ability to determine net MN-PCE induction. This calculation subtracts the frequencies of MN-PCE prior to treatment from the frequencies following treatment, thereby eliminating baseline MN-PCE variability among treatment groups at time 0 (Table 1). Data represent “the absolute value” of the averages of MN-PCE frequencies and were analyzed as follows:

(1) NIF=average  of  MN-PCE  frequencies  measured  at  time  xi−average  of  MN-PCE  frequencies  measured  at  time  0,

where *x*_*i*_ is evaluation at 24, 48, or 72 h per group and time 0 is evaluation at 0 h (before treatment) per group.

**Figure 3:**
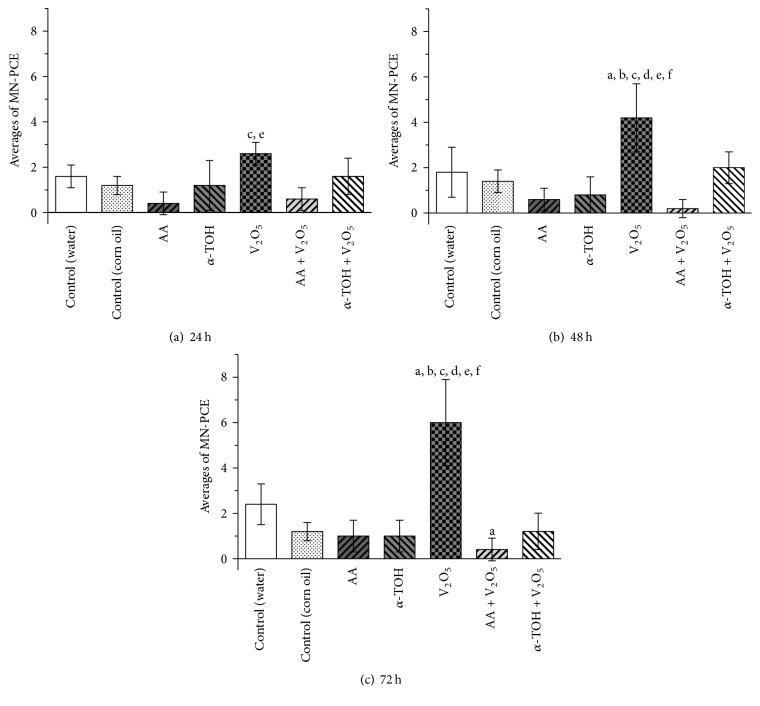
Effect of antioxidants (AA and *α*-TOH) on the MN-PCE NIF in peripheral blood of mice at different times: (a) 24 h, (b) 48 h, and (c) 72 h after treatment with V_2_O_5_. Data represent “the absolute value” of the averages of MN-PCE frequencies obtained at 24, 48, and 72 h per group minus the averages of MN-PCE frequencies at 0 h per group. ANCOVA: ^a^ *p* < 0.05 versus control (water); ^b^ *p* < 0.05 versus control (corn oil); ^c^ *p* < 0.05 versus AA; ^d^ *p* < 0.05 versus *α*-TOH; ^e^ *p* < 0.05 versus AA + V_2_O_5_; ^f^ *p* < 0.05 versus *α*-TOH + V_2_O_5_. 2,000 PCE were evaluated in each mouse (5 mice per group).

Data represent the average MN-PCE frequencies at 24, 48, and 72 h per group, minus the average MN-PCE frequencies at 0 h per group.  Figure 3 shows the NIF of MN-PCE values for all groups at 24, 48, and 72 h after treatment. The frequencies of MN-PCE in samples from the group treated with AA were lower than the control at 24, 48, and 72 h (75, 67, and 58% reduction, resp.) after treatment. In the groups that combined treatments with antioxidants and V_2_O_5_, a significant reduction in the frequencies of MN-PCE was detected: the AA reduced by 77% at 24 h and a complete reduction was observed at 48 and 72 h, while *α*-TOH reduced by 38, 52, and 80% at 24, 48, and 72 h, respectively.

PCE/NCE ratio is shown in Table 2. Treatments with antioxidants and antioxidants + V_2_O_5_ decreased the frequencies of PCE compared to controls. This decrease was more significant when antioxidants were administered alone. Treatment of V_2_O_5_ did not affect the average MN-PCE in the mice (Table 2). The cytotoxic effects were simultaneously assessed by apoptosis, necrosis, and cell viability directly in nucleated cell of peripheral blood of mice at 48 h after treatment.

**Figure 4:**
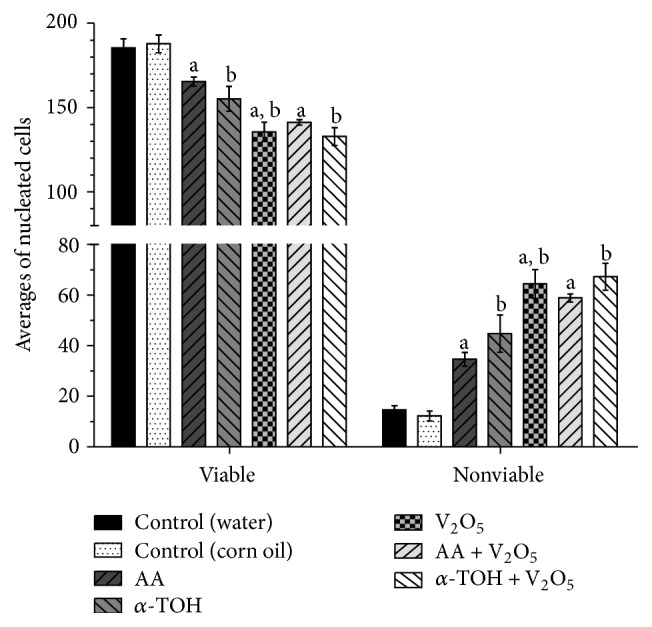
Evaluations at 48 h in peripheral blood of mice. Averages of viable and nonviable nucleated cells. ^a^ *p* < 0.05 versus control (water); ^b^ *p* < 0.05 versus control (corn oil); ^c^ *p* < 0.05 versus AA + V_2_O_5_; ^d^ *p* < 0.05 versus *α*-TOH + V_2_O_5_. Nonviable cells include apoptotic and necrotic cells. 200 nucleated cells were evaluated in each mouse (5 mice per group).

Unlike the results obtained in the PCE/NCE ratio, cell viability also decreased in treatment with V_2_O_5_ alone, which was more significant than in the other treatments (excluding treatment with *α*-TOH + V_2_O_5_) (Figure 4). All treatments significantly increased apoptotic cell frequency, with the highest increases in the V_2_O_5_ and combined groups. Late apoptotic cells were mainly identified in antioxidant and antioxidants + V_2_O_5_ treatments. Lower average early apoptotic cells were found in the treatments with antioxidants compared to those in the control group, and this reduction was significant in the treatment with AA. Although in the AA-treated group the decrease in early apoptotic cells was statistically significant, the increase in late apoptotic cells was significant (Table 3). In the V_2_O_5_ and combined treatments, an increase in both early and late apoptotic cells was observed, being greater in the late apoptotic cells. The necrotic cells increased significantly only in the treatment with V_2_O_5_ alone (Table 3).

No mice exposed to V_2_O_5_ died, and no clinical signs of toxicity were observed.

## 4. Discussion

Although V_2_O_5_ is considered a possible carcinogen in humans based on evidence of lung carcinogenesis in mice [33], the information regarding the genotoxic potential of V_2_O_5_ in models* in vivo* is limited and inconclusive [7]. In this study we observed that the administration of 40 mg/kg of V_2_O_5_ via ip injection increases the frequencies of MN-PCE in peripheral blood. This is consistent with several studies that also evaluated MN-PCE in experimental animals treated with soluble vanadium compounds (Na_3_VO_4_, SVO_5_, and NH_4_VO_3_) administered orally [34, 35] and particularly by inhalation of V_2_O_5_ in males [36]. However, the maximum increases we observed were around 4/1,000 MN-PCE, which are lower than induction by other metals clearly identified as genotoxic agents such as Cr(VI) [31, 37, 38].

The rodent micronucleus assay is used in regulatory test batteries to predict the carcinogenicity of chemical agents through their ability to produce genotoxicity* in vivo*. If a compound increases MN frequencies it is often regarded as definitive evidence of* in vivo* genotoxicity, making it a probable carcinogen [39, 40]. However, it is important that marginal results in the induction of MN be taken with reservation, since there is evidence that compound-related disturbances in rodent physiology, such as body temperature and erythroblast toxicity, can also modify MN frequencies and increase erythropoiesis by stimulating cell division in bone marrow and peripheral blood [39]. These increases in MN may therefore not be a result of direct, intrinsic genotoxic properties of the agent. For this reason, the EPA Gene-Tox Program and the Collaborative Study Group for the Micronucleus Test have proposed a threshold of 4/1,000 MN-PCE increase to define a compound as a genotoxic agent and a threshold of 7.5/1,000 MN-PCE increase to designate it as a positive control agent [28, 29, 40]. Similarly, a range between 0 and 3 MN-PCE has been proposed for the control group in order to consider individual variation among test subjects. Thus, while we did find higher induction of MN-PCE in the water control group compared to the corn oil control group at all evaluation times, the increase was within this proposed control range (spontaneous micronucleus in PCE from untreated animals) [39, 40].

When assessing the PCE/NCE ratio in the group treated with V_2_O_5_ alone, no changes were observed compared to the control group. The PCE/NCE ratio is suggested by MN assay because it is an indicator of cytotoxicity [40]. However, while finding reduced PCE frequency is indicative of cytotoxic effects, negative results must be interpreted with caution because when toxicity occurs during erythropoiesis, the mechanisms of cell division can be activated and mask the effect [39, 40]. Moreover, it has been observed that vanadium compounds can produce lipid peroxidation in the erythrocyte membrane, leading to hemolysis, which could interfere with the erythroid differentiation process [41, 42]. The effects on erythropoiesis could therefore be related to the marginal increase in MN-PCE observed in mice treated with V_2_O_5_. For this reason, we also assessed cell viability in nucleated peripheral blood cells using the differential AO/EB staining technique. The dual fluorochrome assay is an indicator of cell metabolism and death caused by cell membrane injury [12, 32]. With this analysis, a decrease in the viable cells at 48 h in mice treated with V_2_O_5_ was observed, suggesting a cytotoxic effect. However, cytotoxic effects of V_2_O_5_ have not been found in* in vitro* assays in lymphocytes and human mucosal cells [43] or* in vivo* in bone marrow [33]. Although Rojas-Lemus et al. [36] observed a decrease in cell viability in mice 24 h after inhalation of V_2_O_5_ during acute phase, this did not persist for more than a week in peripheral blood leukocytes.

On the other hand, we observed that the administration of 40 mg/kg of V_2_O_5_ via ip injection increased the frequency of apoptotic cells. The apoptotic activity was indicated by the increased frequencies of early and especially late apoptotic cells 48 h after treatment with V_2_O_5_. Anticancer properties have been attributed to vanadium(V) compounds, and apoptosis has been identified as one of the ways to eliminate tumor cells [7]. Vanadium compounds activate different signaling pathways in normal and cancer cells, acting mainly through inactivation of PTPs and/or activation of PTKs. Activation of cellular signaling pathways converges downstream to cooperatively modulate the transcriptional activity of NF-*κ*B or by the suppression of genes involved in cell cycle regulation, DNA repair, and apoptosis [1, 44, 45]. Although it has been suggested that, in p53-defective rodent cells, such as L5178Y, MN induction may be independent of apoptosis [46], apoptosis may contribute to the elimination of micronucleated cells and hence lead to a marginal induction of MN-PCE when administering V_2_O_5_. However, in the mice treated with V_2_O_5_, counts of necrotic cells increased significantly, leading to inflammatory processes. It has been suggested that it is the combination of oxidative stress, inflammation, and genotoxicity that makes this element a possible carcinogen [47].

Recent studies have shown that vanadium(V) in mice induces genotoxic damage and apoptosis through oxidative stress [7, 8, 48]. The* in vivo* administration of AA or *α*-TOH prior to V_2_O_5_ injection decreased MN-PCE formation compared to V_2_O_5_ alone (Figure 2). The ways in which the antioxidants protect cells against V_2_O_5_-induced genetic damage may be related to its oxide-reductive properties. AA is a potent antioxidant (reducing agent) that is capable of scavenging free radicals of reactive oxygen and nitrogen species that have the potential to damage nucleic acids and promote carcinogenesis [49, 50]. Thus, the combination of antioxidant agents such as AA or *α*-TOH with V_2_O_5_ could protect cells from genetic damage. The genotoxicity of vanadium(V) is due to its reduction by NADH to vanadium(IV), generating ^•^OH [10]. Vanadate reacts with thiols to produce V(IV) and thiyl radicals (vanadyl). During catalysis in the reaction of 2-deoxyguanosine with molecular oxygen, 8-hydroxydeoxyguanosine is formed, causing DNA strand breaks [51]. Thus, ascorbate could react with ROS, quenching and converting them into poorly reactive semidehydroascorbate radicals, which cause no DNA damage [52–54], which is reflected in the reduction of MN-PCE (Figure 2). Vitamin E belongs to the family of lipid-soluble vitamins, of which *α*-TOH is the most active form, and like AA, it is a powerful biological antioxidant that may effectively minimize oxidative stress, lipid peroxidation, and toxic effects of ROS in biological systems [24]. Our data demonstrate that AA and *α*-TOH protected cells against V_2_O_5_-induced genetic damage. The reduction of MN-PCE observed with AA and *α*-TOH was more effective than with the administration of high-antioxidant beverages such as green tea [55], red wine [56], and particularly their antioxidant components such as polyphenols [12, 57]. The particular finding regarding the effects of AA was that it reduced the basal MN-PCE, and that presented the strongest protection against genotoxic damage induced by V_2_O_5_.

Both antioxidants tended to reduce the basal early apoptotic cells, and this effect was significant for the AA group. However, the antioxidants increased late apoptotic cells significantly (Table 3), which could be related to the decrease of MN-PCE observed when antioxidants were administered alone as compared with the control group and its own time 0 of evaluation (when no treatments had yet been administered). Apoptosis is a normal and essential aspect of organ development and remodeling that is initiated at birth and continues throughout life [58]. Thus, apoptosis may play an essential role as a protective mechanism against genotoxic agents by removing genetically damaged cells.

Although numerous reports are available in the literature on the cytotoxic and anticarcinogenic effects of antioxidants in different tumor model systems, the molecular mechanisms underlying the anticarcinogenic potential of antioxidants are not completely understood. Specific forms of vitamin E display apoptotic activity against a wide range of cancer cell types while having little or no effect on normal cell function or viability [59]. Similarly, Naidu [19] demonstrated that ascorbyl stearate inhibited cell proliferation by interfering with the cell cycle, reversing the phenotype and inducing apoptosis in human brain tumor glioblastoma (T98G) cells. Therefore, it has been postulated that the mechanism to explain the chemopreventive potential of antioxidants is their chemical ability to target specific cellular signaling pathways that regulate cellular proliferation and apoptosis [60]. This is consistent with our results, in which the administration of AA or *α*-TOH alone elevated the frequencies of apoptotic cells significantly, and their administration prior to treatment of V_2_O_5_ increased apoptosis even further (Figure 4 and Table 3). The main increases were observed in the late apoptotic cells. The interactions between antioxidants and V_2_O_5_ suggest that their influence is neither additive nor antagonistic (Figure 4 and Table 3). In other studies it was observed that the apoptosis-inducing activity of antioxidants might be synergistically enhanced by a combined treatment with chemopreventive [61] or genotoxic agents [62]. The enhanced induction of apoptosis following a combined treatment suggests that this process may contribute to the elimination of the cells with V_2_O_5_-induced DNA damage (MN-PCE).

Some compounds, including vanadium(V) oxide, have emerged as therapeutic drugs for cancer, since intracellular cascade mechanisms may be involved in causing apoptotic cell death. Low levels of ROS can induce activation of transcription factors, promoting mRNA formation and encoding proteins known to be regulated by vanadium; however, high levels of ROS are cytotoxic to the cells and trigger apoptotic mechanisms. It has therefore been proposed that vanadium compounds be used against malignancies, since their cytotoxic effects against cancer cell lines by generating ROS and Reactive Nitrogen Species have already been shown [63, 64]. The ability to overcome the adverse effects of vanadium compounds during therapeutic action is thus a crucial issue for its future use in medicine [64]. In addition, the low costs of vanadium-based drugs make the use of vanadium compounds very promising. Our findings strongly suggest that both AA and *α*-TOH can be used effectively in therapy either alone (antioxidants) or in combination with other agents like V_2_O_5_ to reduce its genotoxicity. Additional studies are required to determine the specific intracellular sites of action that these antioxidants target in order to fully understand the specific mechanisms of action mediating their antigenotoxic and apoptotic effects, as well as to further clarify their potential value as chemotherapeutic agents in the prevention and treatment of diseases related with genotoxic damage, including some cancers.

## Figures and Tables

**Table 1:** Averages of the MN-PCE induction in peripheral blood of mice.

Treatment	Dose (mg/kg)	*N*	Time analysis (hours)	MN-PCE/1,000 cells^∧^ (mean ± SD)	ANOVA
Control (water)	0	5	0	0.5 ± 0.3	
			24	0.8 ± 0.2	
			48	0.9 ± 0.4	
			72	1.2 ± 0.2	

Control (corn oil)	0	5	0	0.2 ± 0.2	
			24	0.3 ± 0.3	
			48	0.4 ± 0.4	
			72	0.3 ± 0.3	

AA^*∗*^	100	5	0	0.5 ± 0.3	
			24	0.2 ± 0.2	
			48	0.3 ± 0.2	
			72	0.3 ± 0.4	

*α*-TOH^*∗*^	20	5	0	0.3 ± 0.4	
			24	0.5 ± 0.3	
			48	0.3 ± 0.2	
			72	0.3 ± 0.2	

V_2_O_5_	40	5	0	1.4 ± 0.4	
			24	2.2 ± 0.2	a, c, d
			48	3.0 ± 0.6	a, b, c, d
			72	3.9 ± 0.8	a, b, c, d

AA + V_2_O_5_	100 + 40	5	0	1.1 ± 0.2	
			24	0.9 ± 0.4	
			48	0.7 ± 0.2	
			72	0.8 ± 0.4	

*α*-TOH + V_2_O_5_	20 + 40	5	0	0.7 ± 0.4	
			24	1.0 ± 0.3	
			48	1.2 ± 0.2	
			72	0.8 ± 0.2	

^a^ *p* < 0.05 versus controls; ^b^ *p* < 0.05 versus V_2_O_5_ 0 h; ^c^ *p* < 0.05 versus AA + V_2_O_5_; ^d^ *p* < 0.05 versus  *α*-TOH + V_2_O_5_.

^*∗*^AA: vehicle water, distilled; *α*-TOH: vehicle corn oil.

^∧^2,000 PCE were evaluated in each mouse (5 mice per group).

**Table 2:** Averages of the PCE/NCE ratio in peripheral blood of mice.

Treatment	Dose (mg/kg)	*N*	Time analysis (hours)	PCE/NCE 1,000 cells^∧^ (mean ± SD)	ANOVA
Control (water)	0	5	0	127.4 ± 5.9	
			24	123.2 ± 11.2	
			48	133.6 ± 10.1	
			72	128.8 ± 4.9	

Control (corn oil)	0	5	0	115.0 ± 3.9	
			24	115.5 ± 4.0	
			48	111.7 ± 3.4	
			72	114.0 ± 2.2	

AA^*∗*^	100	5	0	100.6 ± 2.0	
			24	68.8 ± 1.9	a, b
			48	67.2 ± 3.0	a, b
			72	68.2 ± 0.8	a, b

*α*-TOH^*∗*^	20	5	0	103.4 ± 3.6	
			24	75.4 ± 3.4	a, c
			48	66.2 ± 7.0	a, c
			72	67.8 ± 2.4	a, c

V_2_O_5_	40	5	0	123.8 ± 6.6	
			24	125.4 ± 4.2	d, f
			48	124.8 ± 4.3	d, f
			72	136.6 ± 3.0	d, f

AA + V_2_O_5_	100 + 40	5	0	101.2 ± 4.7	
			24	95.8 ± 5.3	a
			48	88.4 ± 1.3	a
			72	87.6 ± 1.9	a

*α*-TOH + V_2_O_5_	20 + 40	5	0	116.0 ± 4.4	
			24	87.0 ± 3.4	a, e
			48	84.6 ± 3.4	a, e
			72	87.4 ± 2.2	a, e

^a^ *p* < 0.05 versus control; ^b^ *p* < 0.05 versus AA 0 h; ^c^ *p* < 0.05 versus *α*-TOH 0 h; ^d^ *p* < 0.05 versus AA + V_2_O_5_; ^e^ *p* < 0.05 versus AA + V_2_O_5_ 0 h; ^f^ *p* < 0.05 versus *α*-TOH + V_2_O_5_.

^*∗*^AA: vehicle water, distilled; *α*-TOH: vehicle corn oil.

^∧^1,000 erythrocytes were evaluated in each mouse (5 mice per group).

**Table 3:** Evaluations at 48 h in peripheral blood of mice. Averages of early and late apoptotic and necrotic cells per group.

Group	Dose (mg/kg)	*N*	$\overset{-}{x}$ ± SD		
			Early apoptotic	Late apoptotic	Necrotic
Control (water)	0	5	12.6 ± 1.7	1.2 ± 0.4	0.8 ± 0.4
Control (corn oil)	0	5	10.6 ± 2.1	1.0 ± 0.0	0.6 ± 0.5
AA	100	5	1.0 ± 0.7^a^	33.4 ± 2.5^a^	0.2 ± 0.4
*α*-TOH	20	5	6.8 ± 3.2	37.8 ± 4.1^b^	0.2 ± 0.4
V_2_O_5_	40	5	21.2 ± 5.6^a,b^	40.8 ± 5.4^a,b^	2.4 ± 1.3^a,b,c,d^
AA + V_2_O_5_	100 + 40	5	23.8 ± 2.4^a^	34.2 ± 1.9^a^	0.8 ± 0.5
*α*-TOH + V_2_O_5_	20 + 40	5	25.0 ± 3.5^b^	41.6 ± 4.6^b^	0.6 ± 0.5

^a^ *p* < 0.05 versus control (water); ^b^ *p* < 0.05 versus control (corn oil); ^c^ *p* < 0.05 versus AA + V_2_O_5_; ^d^ *p* < 0.05 versus *α*-TOH + V_2_O_5_. Nonviable cells include apoptotic and necrotic cells. 200 nucleated cells were evaluated in each mouse (5 mice per group).

## Abbreviations

*α*-TOH: Alpha-tocopherol
AO: Acridine orange
AA: Ascorbic acid
EB: Ethidium bromide
FDA: Food and Drug Administration
ip: Intraperitoneal
MN: Micronucleus
MN-PCE: Micronucleated polychromatic erythrocytes
NCE: Normochromatic erythrocytes
NIF: Net induction frequency
^•^OH: Hydroxyl radical
PCE: Polychromatic erythrocytes
ROS: Reactive oxygen species
V_2_O_5_: Vanadium pentoxide.

## References

[1] Evangelou A. M. (2002). Vanadium in cancer treatment. *Critical Reviews in Oncology/Hematology*.

[2] Mukherjee B., Patra B., Mahapatra S., Banerjee P., Tiwari A., Chatterjee M. (2004). Vanadium—an element of atypical biological significance. *Toxicology Letters*.

[3] International Agency for Research on Cancer (IARC) (2006). *Cobalt in Hard Metals and Cobalt Sulfate, Gallium Arsenide, Indium Phosphide and Vanadium Pentoxide*.

[4] American Conference of Governmental Industrial Hygienists (ACGIH) (2012). Vanadium pentoxide: chemical substances 7th edition documentation, 9. *Appendix B: Threshold Limit Values (TLVs®) and Biological Exposure Indices (BEIs®)*.

[5] Starr T. B., MacGregor J. A., Ehman K. D., Nikiforov A. I. (2012). Vanadium pentoxide: use of relevant historical control data shows no evidence for a carcinogenic response in F344/N rats. *Regulatory Toxicology and Pharmacology*.

[6] Assem F. L., Levy L. S. (2009). A review of current toxicological concerns on vanadium pentoxide and other vanadium compounds: gaps in knowledge and directions for future research. *Journal of Toxicology and Environmental Health, Part B: Critical Reviews*.

[7] Assem F. L., Oskarsson A. (2015). Vanadium. *Handbook on the Toxicology of Metals*.

[8] Soriano-Agueda L. A., Ortega-Moo C., Garza J., Guevara-García J. A., Vargas R. (2016). Formation of reactive oxygen species by vanadium complexes. *Computational and Theoretical Chemistry*.

[9] Mailhes J. B., Hilliard C., Fuseler J. W., London S. N. (2003). Vanadate, an inhibitor of tyrosine phosphatases, induced premature anaphase in oocytes and aneuploidy and polyploidy in mouse bone marrow cells. *Mutation Research*.

[10] Shi X., Dalal N. S. (1992). Hydroxyl radical generation in the NADH/microsomal reduction of vanadate. *Free Radical Research*.

[11] Costa W. F., Nepomuceno J. C. (2006). Protective effects of a mixture of antioxidant vitamins and minerals on the genotoxicity of doxorubicin in somatic cells of *Drosophila melanogaster*. *Environmental and Molecular Mutagenesis*.

[12] García-Rodríguez M. D. C., Carvente-Juárez M. M., Altamirano-Lozano M. A. (2013). Antigenotoxic and apoptotic activity of green tea polyphenol extracts on hexavalent chromium-induced DNA damage in peripheral blood of CD-1 mice: analysis with differential acridine orange/ethidium bromide staining. *Oxidative Medicine and Cellular Longevity*.

[13] Antunes L. M., Takahashi C. S. (1998). Effects of high doses of vitamins C and E against doxorubicin-induced chromosomal damage in Wistar rat bone marrow cells. *Mutation Research*.

[14] Siddique Y. H., Beg T., Afzal M. (2005). Antigenotoxic effects of ascorbic acid against megestrol acetate-induced genotoxicity in mice. *Human and Experimental Toxicology*.

[15] Singh M., Kaur P., Sandhir R., Kiran R. (2008). Protective effects of vitamin E against atrazine-induced genotoxicity in rats. *Mutation Research—Genetic Toxicology and Environmental Mutagenesis*.

[16] Cameron E., Pauling L. (1979). *Cancer and Vitamin C*.

[17] Block G. (1991). Vitamin C and cancer prevention: the epidemiologic evidence. *The American Journal of Clinical Nutrition*.

[18] Frei B. (1994). Reactive oxygen species and antioxidant vitamins: mechanisms of action. *The American Journal of Medicine*.

[19] Naidu K. A. (2003). Vitamin C in human health and disease is still a mystery? An overview. *Nutrition Journal*.

[20] Sugiyama M., Tsuzuki K., Matsumoto K., Ogura R. (1992). Effect of vitamin E on cytotoxicity, DNA single strand breaks, chromosomal aberrations, and mutation in Chinese hamster V-79 cells exposed to ultraviolet-B light. *Photochemistry and Photobiology*.

[21] Lunec J., Halligan E., Mistry N., Karakoula K. (2004). Effect of vitamin E on gene expression changes in diet-related carcinogenesis. *Annals of the New York Academy of Sciences*.

[22] Packer L. (1991). Protective role of vitamin E in biological systems. *American Journal of Clinical Nutrition*.

[23] Kayden H. J., Traber M. G. (1993). Absorption, lipoprotein transport, and regulation of plasma concentrations of vitamin E in humans. *Journal of Lipid Research*.

[24] Ogutcu A., Uzunhisarcikli M., Kalender S., Durak D., Bayrakdar F., Kalender Y. (2006). The effects of organophosphate insecticide diazinon on malondialdehyde levels and myocardial cells in rat heart tissue and protective role of vitamin E. *Pesticide Biochemistry and Physiology*.

[25] Haber S. L., Wissler R. W. (1962). Effect of vitamin E on carcinogenicity of methylcholanthrene.. *Experimental Biology and Medicine*.

[26] García-Rodríguez M., Serrano-Reyes G., Altamirano-Lozano M. (2012). Comparative study in vivo of the genotoxic damage induced by CrO_3_ and the effects of the antioxidants: ascorbic acid, alfa-tocopherol and beta-carotene. *Free Radical Biology and Medicine*.

[27] Altamirano-Lozano M. A., Montaño-Rodríguez A. R., García-Cárdenas G. P., Peralta-García P., García-Rodríguez M. C. (2013). Estudio de las frecuencias de micronúcleos en sangre periférica de ratón CD-1 tratados con trióxido de cromo, sulfato de talio y pentóxido de vanadio in vivo. *Revista Internacional de Contaminación Ambiental*.

[28] Heddle J. A., Hite M., Kirkhart B. (1983). The induction of micronuclei as a measure of genotoxicity. A report of the U.S. Environmental Protection Agency Gene-Tox Program. *Mutation Research*.

[29] CSGMT (The Collaborative Study Group for the Micronucleus Test) (1995). Protocol recommended by the CSGMT/JEMS.MMS for the short-term mouse peripheral blood micronucleus test. The Collaborative Study Group for the Micronucleus Test (CSGMT) (CSGMT/JEMS.MMS, The Mammalian Mutagenesis Study Group of the Environmental Mutagen Society of Japan). *Mutagenesis*.

[30] Hayashi M., Morita T., Kodama Y., Sofuni T., Ishidate M. (1990). The micronucleus assay with mouse peripheral blood reticulocytes using acridine orange-coated slides. *Mutation Research Letters*.

[31] García-Rodríguez M. C., López-Santiago V., Altamirano-Lozano M. (2001). Effect of chlorophyllin on chromium trioxide-induced micronuclei in polychromatic erythrocytes in mouse peripheral blood. *Mutation Research/Genetic Toxicology and Environmental Mutagenesis*.

[32] McGahon A. J., Martin S. J., Bissonnette R. P. (1995). The end of the (cell) line: methods for the study of apoptosis *in vitro*. *Methods in Cell Biology*.

[33] NTP (2002). Toxicology and carcinogenesis studies of vanadium pentoxide. *NTP TR*.

[34] Ciranni R., Antonetti M., Migliore L. (1995). Vanadium salts induce cytogenetic effects in in vivo treated mice. *Mutation Research*.

[35] Leopardi P., Villani P., Cordelli E., Siniscalchi E., Veschetti E., Crebelli R. (2005). Assessment of the in vivo genotoxicity of vanadate: analysis of micronuclei and DNA damage induced in mice by oral exposure. *Toxicology Letters*.

[36] Rojas-Lemus M., Altamirano-Lozano M., Fortoul T. I. (2014). Sex differences in blood genotoxic and cytotoxic effects as a consequence of vanadium inhalation: micronucleus assay evaluation. *Journal of Applied Toxicology*.

[37] García-Rodríguez M. C., García-Cárdenas G. P., Montaño-Rodríguez A. R., Altamirano-Lozano M. A. (2014). Cytotoxic and genotoxic effects of exposure to heavy metals (chromium *[*VI*]* and thallium *[*I*]*) of mice CD-1 strain: micronucleus, apoptosis and cell viability. *Acta Universitaria*.

[38] EPA. Environmental Protection Agency (2010). Toxicological review of hexavalent chromium. *Support of Summary Information on the Integrated Risk information System (IRIS)*.

[39] Tweats D. J., Blakey D., Heflich R. H. (2007). Report of the IWGT working group on strategies and interpretation of regulatory in vivo tests. I. Increases in micronucleated bone marrow cells in rodents that do not indicate genotoxic hazards. *Mutation Research/Genetic Toxicology and Environmental Mutagenesis*.

[40] Krishna G., Hayashi M. (2000). In vivo rodent micronucleus assay: protocol, conduct and data interpretation. *Mutation Research*.

[41] Hogan G. R. (1990). Peripheral erythrocyte levels, hemolysis and three vanadium compounds. *Experientia*.

[42] Aguirre M. V., Juaristi J. A., Alvarez M. A., Brandan N. C. (2005). Characteristics of in vivo murine erythropoietic response to sodium orthovanadate. *Chemico-Biological Interactions*.

[43] Kleinsasser N. H., Dirschedl P., Staudenmaier R., Harréus U. A., Wallner B. C. (2003). Genotoxic effects of vanadium pentoxide on human peripheral lymphocytes and mucosal cells of the upper aerodigestive tract. *International Journal of Environmental Health Research*.

[44] Manning F. C. R., Patierno S. R. (1996). Apoptosis: inhibitor or instigator of carcinogenesis?. *Cancer Investigation*.

[45] Assimakopoulos D., Kolettas E., Zagorianakou N., Evangelou A., Skevas A., Agnantis N. J. (2000). Prognostic significance of p53 in the cancer of the larynx. *Anticancer Research*.

[46] Whitwell J., Smith R., Jenner K. (2015). Relationships between p53 status, apoptosis and induction of micronuclei in different human and mouse cell lines in vitro: implications for improving existing assays. *Mutation Research/Genetic Toxicology and Environmental Mutagenesis*.

[47] Fortoul T. I., Rodriguez-Lara V., González-Villalva A. (2014). Inhalation of vanadium pentoxide and its toxic effects in a mouse model. *Inorganica Chimica Acta*.

[48] Huang C., Zhang Z., Ding M. (2000). Vanadate induces p53 transactivation through hydrogen peroxide and causes apoptosis. *The Journal of Biological Chemistry*.

[49] Crott J. W., Fenech M. (1999). Effect of vitamin C supplementation on chromosome damage, apoptosis and necrosis *ex vivo*. *Carcinogenesis*.

[50] Mamede A. C., Tavares S. D., Abrantes A. M., Trindade J., Maia J. M., Botelho M. F. (2011). The role of vitamins in cancer: a review. *Nutrition and Cancer*.

[51] Shi X., Jiang H., Mao Y., Ye J., Saffiotti U. (1996). Vanadium(IV)-mediated free radical generation and related 2′-deoxyguanosine hydroxylation and DNA damage. *Toxicology*.

[52] Putchala M. C., Ramani P., Sherlin H. J., Premkumar P., Natesan A. (2013). Ascorbic acid and its pro-oxidant activity as a therapy for tumours of oral cavity—a systematic review. *Archives of Oral Biology*.

[53] Crans D. C., Baruah B., Gaidamauskas E., Lemons B. G., Lorenz B. B., Johnson M. D. (2008). Impairment of ascorbic acid's anti-oxidant properties in confined media: inter and intramolecular reactions with air and vanadate at acidic pH. *Journal of Inorganic Biochemistry*.

[54] Horton D. C., VanDerveer D., Krzystek J. (2014). Spectroscopic characterization of *L*-ascorbic acid-induced reduction of vanadium(V) dipicolinates: formation of vanadium(III) and vanadium(IV) complexes from vanadium(V) dipicolinate derivatives. *Inorganica Chimica Acta*.

[55] García-Rodríguez M. C., Vilches-Larrea R. E., Nicolás-Mendez T., Altamirano-Lozano M. A. (2012). Green tea and its role on chemoprevention in vivo of genotoxic damage induced by carcinogenic metals (chromium [VI]). *Nutricion Hospitalaria*.

[56] García-Rodríguez M. D. C., Mateos-Nava R. A., Altamirano-Lozano M. (2015). *In vivo* effect of red wine undiluted, diluted (75%) and alcohol-free on the genotoxic damage induced by potential carcinogenic metals: chromium [VI]. *Nutrición Hospitalaria*.

[57] García-Rodríguez M. D. C., Nicolás-Méndez T., Montaño-Rodríguez A. R., Altamirano-Lozano M. A. (2014). Antigenotoxic effects of (−)-epigallocatechin-3-gallate (EGCG), quercetin, and rutin on chromium trioxide-induced micronuclei in the polychromatic erythrocytes of mouse peripheral blood. *Journal of Toxicology and Environmental Health Part A*.

[58] Raff M. (1998). Cell suicide for beginners. *Nature*.

[59] Sylvester P. W. (2007). Vitamin E and apoptosis. *Vitamins and Hormones*.

[60] Kerr J. F., Wyllie A. H., Currie A. R. (1972). Apoptosis: a basic biological phenomenon with wideranging implications in tissue kinetics. *British Journal of Cancer*.

[61] Gao Y., Li W., Jia L., Li B., Chen Y. C., Tu Y. (2013). Enhancement of (−)-epigallocatechin-3-gallate and theaflavin-3-3′-digallate induced apoptosis by ascorbic acid in human lung adenocarcinoma SPC-A-1 cells and esophageal carcinoma Eca-109 cells via MAPK pathways. *Biochemical and Biophysical Research Communications*.

[62] García-Rodríguez M. C., Montaño-Rodríguez A. R., Altamirano-Lozano M. A. (2016). Modulation of hexavalent chromium-induced genotoxic damage in peripheral blood of mice by epigallocatechin-3-gallate (EGCG) and its relationship to the apoptotic activity. *Journal of Toxicology and Environmental Health, Part A: Current Issues*.

[63] Kioseoglou E., Petanidis S., Gabriel C., Salifoglou A. (2015). The chemistry and biology of vanadium compounds in cancer therapeutics. *Coordination Chemistry Reviews*.

[64] Pessoa J. C., Etcheverry S., Gambino D. (2015). Vanadium compounds in medicine. *Coordination Chemistry Reviews*.

